# Testicular Tissue Banking for Fertility Preservation in Young Boys: Which Patients Should Be Included?

**DOI:** 10.3389/fendo.2022.854186

**Published:** 2022-03-10

**Authors:** Emily Delgouffe, Aude Braye, Ellen Goossens

**Affiliations:** Biology of the Testis (BITE), Department of Reproduction, Genetics and Regenerative Medicine (RGRG), Vrije Universiteit Brussel (VUB), Brussels, Belgium

**Keywords:** immature testicular tissue banking, prepubertal boys, fertility preservation, male infertility, spermatogonial stem cells, germ cell loss, testis, (non-)malignant disease

## Abstract

Due to the growing number of young patients at risk of germ cell loss, there is a need to preserve spermatogonial stem cells for patients who are not able to bank spermatozoa. Worldwide, more and more clinics are implementing testicular tissue (TT) banking programs, making it a novel, yet indispensable, discipline in the field of fertility preservation. Previously, TT cryopreservation was predominantly offered to young cancer patients before starting gonadotoxic chemo- or radiotherapy. Nowadays, most centers also bank TT from patients with non-malignant conditions who need gonadotoxic conditioning therapy prior to hematopoietic stem cell (HSCT) or bone marrow transplantation (BMT). Additionally, some centers include patients who suffer from genetic or developmental disorders associated with prepubertal germ cell loss or patients who already had a previous round of chemo- or radiotherapy. It is important to note that the surgical removal of TT is an invasive procedure. Moreover, TT cryopreservation is still considered experimental as restoration methods are not yet clinically available. For this reason, TT banking should preferably only be offered to patients who are at significant risk of becoming infertile. In our view, TT cryopreservation is recommended for young cancer patients in need of high-risk chemo- and/or radiotherapy, regardless of previous low-risk treatment. Likewise, TT banking is advised for patients with non-malignant disorders such as sickle cell disease, beta-thalassemia, and bone marrow failure, who need high-risk conditioning therapy before HSCT/BMT. TT retrieval during orchidopexy is also proposed for patients with bilateral cryptorchidism. Since patients with a medium- to low-risk treatment generally maintain their fertility, TT banking is not advised for this group. Also for Klinefelter patients, TT banking is not recommended as it does not give better outcomes than a testicular sperm extraction later in life.

## Introduction

Over the last decades, the overall rate of childhood and adolescent cancer incidence has increased. The death rates, on the other hand, have strongly decreased due to major advances in oncological treatments. Nowadays, long-term survival is expected in about 80% of young cancer patients (1). In Europe, about 35,000 new cases of childhood and adolescent cancer are reported each year, leading to nearly half a million cancer survivors in 2020 (2). Because of the increase in childhood cancer survivors, an increasing number of adults will have to deal with the long-term effects of cancer treatments. A major complication is the damage to the germ cells and testicular somatic cells caused by the gonadotoxic properties of chemo- and/or radiotherapy. Depending on the survival and functionality of these cells, the testicular damage can result in temporary or even permanent infertility (3). The severity of the testicular damage depends on the type and cumulative dose of chemotherapeutics, the duration, site of irradiation, and fractionation schedule of radiotherapy (4–6). In addition, the patient’s age and his individual sensitivity to the gonadotoxic treatment will further determine the fertility outcome (7). Nowadays, gonadotoxic treatments are also used as conditioning therapy for children with non-malignant disorders (e.g., sickle cell disease (SCD) and thalassemia) in need of a bone marrow transplantation (BMT) or hematopoietic stem cell transplantation (HSCT) (8). To prevent infertility, fertility preservation is often recommended before starting high-risk gonadotoxic treatment in both malignant and non-malignant patients.

The first line method in male fertility preservation is the collection and cryopreservation of mature spermatozoa. However, this method is only applicable when the patient can produce a sperm sample. For pubertal patients with active spermatogenesis who cannot provide a sperm sample by masturbation, assisted ejaculation techniques such as penile vibratory stimulation or electroejaculation under general anesthesia can be used to retrieve sperm. Alternatively, sperm can be obtained from the testicle *via* testicular sperm extraction (TESE) (9). If these techniques are not successful, or not possible because spermatogenesis did not start yet, testicular tissue (TT) can be harvested and banked to preserve the spermatogonial stem cells (SSCs) (10). For prepubertal patients, TT banking is the only available option for fertility preservation.

Since the first experimental fertility preservation program for (pre)pubertal boys in 2002, more centers around the world have started to collect and cryopreserve immature TT from boys at risk of losing their SSCs (10). An international survey conducted in 2019 showed that testicular tissue from more than 1,000 (pre)pubertal boys was banked, which is a 4-fold increase compared to a first survey in 2012 (10, 11). This finding highlights the increasing efforts and acceptability of immature TT cryopreservation programs. The 2019 survey also demonstrated that the indications for TT banking have been expanding since 2012. Previously, most centers almost exclusively offered TT banking to young cancer patients before their cancer treatment (10). Today, the majority also includes patients with non-malignant diseases at risk of germ cell loss, and some include patients who already had a previous round of chemo- and/or radiotherapy. TT banking is also offered to patients with certain genetic or testicular disorders (e.g., Klinefelter syndrome (KS) and cryptorchidism) that are associated with testicular failure leading to fertility problems (11).

The three restoration methods that are currently under development are 1) auto-transplantation of the thawed TT, 2) auto-transplantation of isolated SSCs from the TT, and 3) *in vitro* spermatogenesis (11, 12). Recently, offspring were achieved in non-human primates after successful prepubertal TT grafting (13). When translating this technique to the clinic, the risk of reintroducing tumor cells should be taken into account. Therefore, this method is not recommended for patients who suffered from childhood hematological cancers or metastatic malignancies. For these patients, SSC propagation and auto-transplantation of SSCs would be a better option as the malignant cells could be eliminated during the cell sorting process (7). Although non-human primate embryos (14) and two generations of healthy mouse offspring (15, 16) were obtained after SSC transplantation, the SSC sorting steps and propagation still need further optimization before clinical application is possible. Alternatively, *in vitro* spermatogenesis is proposed to avoid reintroducing malignant cells in a cured patient (11). However, to date, complete *in vitro* spermatogenesis is only achieved in rodents (17) and further culture improvements are needed to achieve safe and efficient human *in vitro* spermatogenesis (18).

Despite the progress that has been made over the past few years, TT banking remains an experimental method as these fertility restoration techniques are currently not yet implemented in clinical practice (19, 20). Moreover, to collect TT, the patients must undergo an invasive biopsy procedure. Although this procedure is shown to have a low short-term complication risk of 2-3% (21–25), only a few studies investigated the possible adverse effects in the longer term. In the prospective study of Uijldert et al. from 2017 (23), it was shown in 64 boys that, up to one year after surgery, the testicular growth of the biopsied testis was similar to the contralateral non-biopsied testis. This finding was confirmed in the prospective study of Borgström et al. (26) in which 21 (pre)pubertal boys, who underwent a TT biopsy before HSCT, were followed for 5.0 to 13.7 years. During this follow-up, the follicle-stimulating hormone (FSH), luteinizing hormone (LH), and testosterone levels were within normal limits for age and pubertal stage in the fourteen surviving patients. However, at the last visit, nine showed high FSH levels, and three showed high LH levels. Besides this, seven out of nine patients showed subnormal anti-Müllerian hormone (AMH) and inhibin B (INHB) levels during the follow-up. This study concluded that these deviating hormone levels were due to the conditioning therapy before HSCT rather than the biopsy procedure itself. Therefore, they concluded that the biopsy procedure had no long-term risks. Reproductive hormone levels were also analyzed in a retrospective cohort study by Kanbar et al. (27). Of the 57 (pre)pubertal boys who underwent TT banking before or after gonadotoxic treatment initiation, 37% had abnormal gonadotropin levels, 4% had hypogonadotropic hypogonadism, and 33% had primary gonadal failure. These results are in line with previous reports on hypogonadism in childhood cancer survivors (28–31). Therefore, they concluded that a unilateral TT biopsy of <5% of tissue during childhood does not appear to adversely affect the reproductive health outcomes. Despite these reassuring first results, more long-term prospective case-control studies are needed to guarantee the safety of the TT banking procedure.

All taken together, TT banking remains a relatively new technique of which many aspects still require further investigation. To safeguard the well-being of the young patients, and to avoid unnecessary surgery, strict selection criteria are required for this procedure. In this review, the risk of infertility for the most frequent conditions and treatments will be described based on the most recent findings. Moreover, as the indications for TT banking vary between centers, this review aims to revise the current inclusion criteria and gives advice on which patients are (still) eligible for TT banking.

## Malignant Diseases

The most common indication for TT banking continues to be childhood malignant disease. Within this category, the most prominent disorders are hematological cancers, central nervous system tumors, Ewing family tumors, rhabdomyosarcoma, osteosarcoma, neuroblastoma, and other non-central nervous system solid tumors (11). Malignancies as testicular cancer, extragonadal germ cell tumors, Hodgkin’s disease, and leukemia are associated with a reduced gonadal function, even before the start of the treatment (32–40). The reproductive health of cancer patients can be affected through various mechanisms. Firstly, the cancer itself can disrupt the hypothalamic-pituitary-gonadal (HPG) axis in the patient, which is fundamental for the reproductive hormone production and later fertility. Some tumors produce endocrine substances like beta-human chorionic gonadotropin or alpha-fetoprotein, that trigger negative feedback on the HPG axis (41–44). Other cancers like leukemia, lymphoma, or central nervous system tumors directly impair the pituitary gland through tumor cell invasion (45) Secondly, some tumors directly impair the testicular function by invading the healthy testicular tissue and causing local damage to the cells (5). Thirdly, the pro-inflammatory response and other effects of the malignancy (e.g., pain, fever, and anorexia) may also impair the patient’s fertility (34, 36, 45, 46). Lastly, the cancer diagnosis itself may affect the psychological wellbeing of the patient, potentially causing an additional negative impact on spermatogenesis and sexual function in adolescence and adulthood (47–49).

Although the malignancy itself plays a role, the fertility decline in cancer patients predominantly depends on their treatment with surgery, chemotherapy, local or total body irradiation, and/or a combination of those. The cancer treatment can either cause direct damage to the germ cells and/or the somatic cell populations of the testis (3), or it can have indirect effects through damage to the HPG axis (50). Contrary to previous findings, recent evidence indicates that gonadotoxic therapy can impair the gonadal function at any pubertal stage (51, 52). It has even been shown that the prepubertal testis is more susceptible to the damaging effects of gonadotoxic agents than adults because of the constant turnover of Sertoli cells and early germ cells (53, 54). Production of mature spermatozoa during puberty is determined by the number of surviving SSCs and the functionality of the somatic cell populations. A drastically reduced SSC pool may require several years before spermatogenesis recovers. However, a completely depleted SSC pool will result in permanent infertility (3, 55, 56). The risk for infertility depends on the type and dose of administered agents and the combination of the received cytotoxic therapy (7). The major cytotoxic treatment regimens are therefore categorized into low-, medium- and high-risk groups (5, 57, 58). The degree of gonadal damage for the different treatment modalities will be discussed in the following sections.

### Surgery

Tumor resection surgery involving the reproductive organs can affect the patient’s fertility potential. Performing a unilateral orchiectomy in patients with testicular cancer results in decreased germ cell numbers, and may put them at risk for prematurely reduced Leydig cell function (59–61). In these patients, both the cancer itself and the surgical intervention can cause impaired spermatogenesis (62). Most types of central nervous system tumors require tumor resection, which may cause damage to the hypothalamus and/or pituitary glands, leading to gonadotropin deficiencies (63). In some cases, chemo- and/or radiotherapy can be used to circumvent a surgical intervention in the gonads or hypothalamus-pituitary region. However, these treatments could in turn also increase the risk for gonadal damage (1).

### Chemotherapy

Chemotherapy is one of the most commonly used treatments to cure cancer. Its principle is to target the rapidly dividing cancer cells. Nevertheless, chemotherapy does not only target the cancerous cells, it also targets other healthy dividing cell populations (64). Sertoli cells are also actively dividing during childhood, making them an additional target for cytotoxic agents (3). Although limited data are available on the long-term impact, Sertoli cell dysfunction was reported following chemotherapy exposure during infancy (65, 66). Leydig cells seem more resistant to chemotherapeutic agents as reports on androgen insufficiency following chemotherapy alone are uncommon (3, 67).

Chemotherapeutic agents can be categorized as low-, medium-, or high-risk depending on their expected degree of gonadal damage. **Table 1** gives an overview of frequently used chemotherapeutic agents and their impact based on previous publications (5–7, 58, 68, 69). The high-risk group consists of high-dose alkylating and platinum-based agents that are associated with a >80% risk of infertility (5, 56, 71). The medium-risk group mainly contains low-dose alkylating and platinum-based agents, anthracyclines, and antimetabolites. The compounds in this group give an estimated risk of 20-80% on infertility (5). The low-risk group consists of vinca alkaloids, certain antimetabolites, and non-anthracycline antibiotics (6, 68, 72). Low-risk agents generally induce a short-term interruption of spermatogenesis with a <20% risk of prolonged azoospermia (5).

**Table 1:** Estimated risk of prolonged azoospermia with chemo- and radiotherapy.

	High risk (indication for TT banking)	Medium risk	Low risk
**Chemotherapy**	Busulfan >600 mg/m²	Carboplatin >2 g/m²	Actinomycin-D UD
	(5, 7, 58)	(5, 7, 58, 68)	(5, 7)
	Carmustine 1 g/m²	Cisplatin 400–600 mg/m²	Azathioprine UD
	(5–7, 69)	(5, 7, 58)	(7, 69)
	Chlorambucil >1.4 g/m²	Cyclophosphamide 7.5-19 g/m²	Bleomycin UD
	(5–7, 58, 68, 69)	(5)	(5–7, 58, 68, 69)
	Chlormethine UD	Cytarabine 1 g/m²	Cytarabine <1 g/m²
	(7, 58, 69)	(7, 68)	(5)
	Cisplatin >600 mg/m²	Dacarbazine UD	Dactinomycin UD
	(5–7, 68, 69)	(7, 69)	(58, 68)
	Cyclophosphamide >19 g/m²	Daunorubicin UD	Etoposide UD
	(5–7, 58, 68, 69)	(7, 69)	(5–7, 68, 69)
	Ifosfamide >52 g/m²	Doxorubicin >770 mg/m²	Fludarabine UD
	(5–7, 58, 68)	(5–7, 58, 68, 69)	(7, 69)
	Lomustine 500 mg/m²	Gemcitabine UD	5-Fluoracil UD
	(if treated before puberty) (5, 6)	(7, 69)	(5, 6, 69)
	Mechlorethamine UD	Ifosfamide 42–52 g/m²	6-Mercaptopurine UD
	(7, 68)	(5)	(5–7, 58, 68, 69)
	Melphalan >140 mg/m²	Mitoxantrone UD	Methotrexate UD
	(5–7, 58, 68, 69)	(7, 69)	(5, 6, 58, 68, 69)
	Mustine UD	Oxaliplatin UD	Vinblastine 50 g/m²
	(7)	(7, 68, 69)	(5–7, 58, 68, 69)
	Procarbazine >4 g/m²	Thiotepa 400 mg/m²	Vincristine 8 g/m²
	(5–7, 58, 68, 69)	(5, 7, 69)	(5–7, 58, 68, 69)
**Radiotherapy**	Total body irradiation (5, 7, 58)	Craniospinal- and cranial radiotherapy ≥25 Gy (5, 58, 70)	Lower radiation doses
	Testicular radiotherapy (5, 7, 58, 70)	Scattered abdominal or pelvic radiation ≥1 Gy (5)	

UD, undetermined dose.

It is important to note that high cumulative doses and the combination of different cytotoxic agents can significantly increase the risk for long-term fertility problems (73). Besides this, it remains a challenge to identify the individual risk per molecule, as these are usually administered in combinations (58, 67, 69, 74). Moreover, most data either come from animal experiments or studies performed in adult men, which makes it difficult to extrapolate these risks to young patients (67, 75, 76). As the exact risk classifications/dosages for children remain unclear, further research on prepubertal exposure is warranted together with regular updates of this classification (67, 68).

### Radiotherapy

Another frequently used cancer treatment is radiotherapy. Analogous to chemotherapeutic agents, radiation targets the cancerous cells but has also off-targets like the gonads. The testes are one of the most radiosensitive tissues of the male body (72). Damage induced by radiotherapy is highly dependent on the total dose, duration, the irradiation field, and the fractionation schedule (77). Fractionated radiation usually diminishes the side effects, however, the testicular tissue seems more sensitive to fractionated therapy as it reduces the time available for repair (58, 72). Testicular radiation doses of 0.1-1.2 Gy can result in temporarily oligo- or azoospermia. Doses of 2-3 Gy affect the SSCs, causing long-term infertility in the patient. Doses of 6 Gy and more completely deplete the SSC pool and lead to permanent azoospermia (78–80). Total body irradiation (TBI), used as conditioning therapy before BMT or HSCT, is also linked to gonadal dysfunction (81).

Testicular irradiation and TBI with doses of 10-12 Gy have been reported to harm Sertoli cells. The damage to the Sertoli cell is linked with smaller testicular volumes at adulthood and high FSH and low INHB levels (53, 82, 83). Recent research indicates that Sertoli cells are the most radiosensitive just before the start of spermatogenesis. This is probably due to the high proliferation rate of the Sertoli cells in the testis during early puberty (3, 82, 84). Leydig cells are generally more resistant to irradiation damage. Leydig cell dysfunction and testosterone deficiency are usually undetected up to doses of 30 Gy in adult men and 20 Gy in prepubertal boys (4, 73, 85). This might be attributed to a compensatory increase in LH levels that corrects testosterone production, leading to a normal puberty initiation despite the gonadal damage (3, 58).

Impaired fertility has also been reported after cranial radiotherapy using doses of 35–45 Gy. Cranial radiotherapy does not directly damage the gonads, but it is known to cause neuro-endocrine imbalances when the HPG axis lies within the irradiation field (86). This might cause gonadotropin deficiencies, leading to decreased sex steroid levels and resulting in impaired spermatogenesis and delayed puberty (87). When the gonadotropin deficiency is only caused by damage to the HPG axis, it can be treated by administration of exogenous gonadotropin-releasing hormone (GnRH) or gonadotropins (58). However, when there is additional gonadal damage, the problem could persist (88). Cranial radiotherapy with doses of 25 Gy and more can, paradoxically, also cause early puberty by prematurely activating the HPG axis. This can be resolved by the administration of GnRH analogs (89).

As shown in **Table 1**, a classification can be made based on the risk for later fertility problems. The high-risk class (>80%) consists of TBI and testicular radiotherapy (5, 58). Analogous to chemotherapy, high-risk treatment is restricted as much as possible in current pediatric treatment protocols because of the late adverse effects (1, 90, 91). Some papers only consider testicular irradiation with doses of 6 Gy and more as high-risk (5, 92). Nevertheless, because even very small doses of testicular irradiation can cause irreversible damage to the SSCs (78, 79), any dose should be considered as a risk (58, 70, 73). Craniospinal- and cranial irradiation doses of 25 Gy and more as well as testicular irradiation of 1-6 Gy using scattered abdominal or pelvic radiation should be considered as medium-risk therapy (20-80%) (5, 58). Lower radiotherapy doses are classified as low risk (<20%).

### Targeted Therapy

Targeted cancer treatments are gaining popularity in the field of oncological research and clinical application. In contrast to conventional chemo- and radiotherapy, targeted therapies will specifically act on the cancerous cells instead of all rapidly dividing cells (93). Many targeted cancer therapies are currently being studied in (pre)clinical trials and several have already been approved for specific cancer types by the Food and Drug Administration (FDA) (94). Tyrosine kinase inhibitors, for example, are nowadays indispensable in the treatment of childhood chronic myeloid leukemia and acute lymphoblastic leukemia. Nevertheless, only limited data are available on the impact of targeted therapy on male fertility and more research is needed on this topic (32). The effects of FDA-approved targeted cancer therapies on male fertility that are already known are summarized in a recent review (19). Briefly, the use of Imatinib, Dasatinib, Everolimus, Gemtuzumab, and Voxelotor was associated with some adverse effects on the reproductive health in rat studies. Moreover, Imatinib was found to decrease the sperm count in clinical adult human studies and a case report showed severe oligozoospermia when Imatinib was taken during prepuberty. Interestingly, short-term Imatinib treatment in prepubertal rats did not cause infertility nor affect the offsprings’ health. For Nilotinib, Entrectinib, Larotrectinib, Selumetinib, Avelumab, Nivolumab, Rituximab, Dinutuximab, Blinatumomab, Pembrolizumab, Ipilimumab, Tisagenlecleucel, and Tagraxofusp, no adverse effects on male fertility were found so far. However, more studies on the impact on the prepubertal testis and later spermatogenesis are required to verify these first results.

## Non-Malignant Diseases

Nowadays, HSCT is not only a curative treatment option for patients with malignant hematological diseases, but also for patients with SCD, thalassemia, bone marrow failure, or other non-malignant conditions (11). HSCT requires conditioning therapy with high-risk chemotherapy and/or TBI. Therefore the patients are at risk for long-term side effects such as impaired pubertal development and/or impaired fertility (3, 8, 74, 95–97). In some cases, previous treatment or the disease itself could have caused spermatogonia loss (74). Young boys treated for non-malignant diseases are also suitable candidates for TT banking. The infertility risks for the most prominent diseases will be further elaborated in the following paragraphs.

### Sickle Cell Disease

SCD or sickle cell anemia is the most common non-malignant condition for which allogeneic HSCT is recommended (74). Each year, more than 250,000 children are born with this condition (98). According to the questionnaire of Goossens and colleagues (11), SCD is the main indication for immature TT banking within the benign diseases. SCD is an autosomal recessive disorder in which abnormal hemoglobin S is created that will form polymers when deoxygenated. This gives the red blood cells their characteristic sickle shape, leading to chronic hemolysis and vaso-occlusion (99, 100). Due to tissue hypoxia, multiple organs can be damaged throughout the patient’s life and common complications are severe chronic pain, chronic organ failure, stroke, acute chest syndrome, infections, and early mortality (101–103).

For many years, it is known that the SCD itself can cause delayed puberty as well as disturbed semen parameters and sperm abnormalities at adulthood (104–106). The most common cause for infertility in SCD men is hypogonadism. Furthermore, fertility problems can also be linked to erection disorders, sexual problems, testicular ischemia/infarction, zinc deficiency, and small testicular size (107–109). In addition to the disease itself, many of its primary treatments are associated with fertility impairment. SCD patients are often prescribed opioids and analgesics to ease their chronic pain (99). In general, opioids can decrease the levels of reproductive hormones like LH, testosterone, and estradiol. Chronic opioid use is shown to cause hypogonadism and could also disturb the secretion of other pituitary hormones (110). SCD patients require frequent blood transfusions to control their anemia (99). These transfusions can cause severe iron accumulation, which can be disposed in several organs, including the pituitary gland and testicles. This could cause hypogonadism and damage the testicular cells, leading to impaired fertility (32). To prevent or treat this iron toxicity, patients often take iron chelators to remove the iron excess. However, intensive iron chelation therapy is suspected to suppress spermatogenesis (111).

Hydroxyurea (HU) therapy is another commonly used treatment to prevent vaso-occlusive pain episodes, acute chest syndrome, hospital admissions, and the need for blood transfusions in SCD patients. HU is an FDA-approved non-alkylating antineoplastic agent that can increase the concentration of fetal hemoglobin and thus prevent the sickling of the red blood cells with the mutated adult hemoglobin (107, 112). One of the main consequences of HU is that it seems to worsen the semen parameters in adults (107). To date, only a few studies are available regarding the effect of HU treatment on the number of spermatogonia in prepubertal patients, and the results are contradictory. Some studies have reported that the spermatogonial pool is drastically reduced or even fully depleted following HU treatment (25, 74, 113). More recent studies, however, suggest that HU therapy might not be the main cause of the spermatogonial loss and fertility problems in SCD patients with severe genotypes (114–116). Nevertheless, as the studies on HU did not always include an untreated control group, the effects of SCD itself are difficult to distinguish from those of the HU therapy. Most of the studies only contained a limited number of patients, making it difficult to draw firm conclusions. Larger follow-up studies are needed to verify whether: 1) the spermatogonial loss and disturbed semen parameters are directly caused by the HU treatment or SCD itself or a combination of both, 2) there is a correlation between the timing and duration of HU treatment and the severity of fertility impairment, and, 3) the effects are (partially) reversible after cessation of the HU therapy (107, 108, 115, 117). HU is an antimetabolite classified within the medium-risk group (**Table 1**). For this reason, TT banking is not advised as long as the patient is not eligible for HSCT.

For patients with a severe SCD phenotype and end-organ damage, allogeneic HSCT is recommended (117, 118). This treatment option does involve side effects like risk for rejection, transplant-related mortality, and chronic graft-versus-host disease (115). Certain immunosuppressants that are administered for transplantation are associated with hypogonadism and reduced male fertility (119). Nonetheless, the biggest impact on their fertility is caused by the myeloablative conditioning therapy they have to undergo before HSCT. This conditioning regimen typically consists of high doses of alkylating agents such as cyclophosphamide and busulfan and/or TBI. As summarized in **Table 1**, these treatments all belong to the high-risk category and can cause endocrine dysfunction and impaired fertility (108, 120). Moreover, patients with SCD have been shown to already have a reduced number of spermatogonia before HSCT (74, 103, 114). Because of this, TT banking is recommended for these boys before they start high-risk therapy.

### Beta-Thalassemia

Beta-thalassemia is the second most common hereditary hematological disorder, with about 60,000 new symptomatic cases each year (98). Beta-thalassemia is an autosomal recessive disease in which the production of beta-globin chains, that constitute adult hemoglobin, is disturbed (121). This defect leads to ineffective erythropoiesis and hemolysis in the bone marrow or spleen (122). In beta-thalassemia intermedia (B-TI), the beta-globin chain synthesis is severely reduced. The most severe form of the disease is transfusion-dependent thalassemia, better known as beta-thalassemia major (B-TM). In B-TM patients there is no beta-globin chain synthesis, leading to serious hemolysis, anemia, and hypoxia in several tissues, including the testis (122, 123).

B-TM patients need lifelong blood transfusions to avoid skeleton deformities, hepatomegaly, and splenomegaly (121). As discussed in the SCD section, blood transfusions can initiate iron build-up and severely affect several organs, including the pituitary gland and testicles, causing hypogonadism and testicular tissue damage (32, 123, 124). Moreover, the iron overload is usually worse in B-TM patients as their excessive hemolysis exacerbates the iron release. This is also a reason why B-TM patients are generally more affected than B-TI patients (63, 103). Leptin synthesis can also be decreased in B-TM patients due to iron accumulation in the adipose tissue, further impairing sexual maturation and fertility (125). As for SCD patients, iron chelation therapy can minimize the iron accumulation but may impair spermatogenesis (111). Besides this, decreased seminal and plasma zinc levels are not uncommon in these patients (126). Due to a combination of all the above-mentioned effects, delayed puberty and fertility issues are common findings in B-TM males (103, 124, 126–129).

Despite the declined morbidity and mortality with periodic blood transfusion and iron chelation, HSCT remains the only curative option for patients with B-TM (103). Transplanted patients generally have a better long-term life quality than patients receiving chronic blood transfusions and chelation therapy (123). Nevertheless, transplanted patients are known to develop secondary complications from their high-risk myeloablative conditioning therapy and HSCT, including hypogonadism and infertility, as previously discussed (123, 129). As with SCD, patients with B-TM may have a reduced number of spermatogonia, already before undergoing HSCT (74). Therefore, it is advocated to preserve TT in B-TM patients before they start high-risk conditioning treatment, which depletes the SSC pool even further (124). Previous research showed that more adverse effects are visible in patients who underwent transplantation at an older age (>15 years) or patients who did not receive sufficient chelation therapy before transplantation (130, 131). Nonetheless, since the conditioning therapy still poses a very high risk of infertility, and it is currently impossible to predict with certainty which patients will remain fertile, it is counseled to bank immature TT tissue regardless of age at transplantation.

### Bone Marrow Failure

Childhood bone marrow failure (BMF) is another well-represented group of benign conditions for which immature TT is banked (11). The term BMF encompasses a heterogeneous spectrum of diseases in which the patient has impaired hematopoiesis in the bone marrow, causing inadequate production of one or more circulating blood cell lines (erythroids, myeloids, and/or platelets) (132). BMF can both be acquired (e.g., aplastic anemia and paroxysmal nocturnal hemoglobinuria) or congenital (e.g., Fanconi anemia and dyskeratosis congenita). Aplastic anemia is the most common syndrome on this list (132, 133). Clinical manifestations of these disorders are anemia, neutropenia, and/or thrombocytopenia, and may be associated with hemolysis and thrombophilia (134).

Similar to the previously discussed blood disorders, first-line treatment often consists of blood transfusions to counteract the anemia, as well as chelation therapy to limit iron overload. Platelet transfusion can be administered to prevent or treat bleeding due to thrombocytopenia (132). In the case of acquired aplastic anemia, a condition associated with autoimmunity, immunosuppressive therapy with antithymocyte globulin and cyclosporine A is used (132, 135, 136). So far, this therapy has not been associated with reduced fertility in women, but the exact effect on male fertility has yet to be explored (137). However, as with most benign blood disorders, HSCT can be offered to patients with a severe form of BMF to restore the function of the hematopoietic stem cells. This is often the case in patients with Fanconi anemia or (very) severe aplastic anemia to improve their survival rates (138). The standard conditioning regimen for these diseases contains high-dose cyclophosphamide, inducing a high risk for gonadal dysfunction as shown in **Table 1** (132, 138). Hence, it is advised to store immature TT of these patients before the start of conditioning therapy.

Fanconi anemia often causes genital abnormalities as cryptorchidism, hypospadias, seminiferous tubule hypoplasia, and small testes. The disease itself is also shown to affect the HPG-axis, with hypogonadism, delayed or accelerated puberty, and fertility issues as previously discussed (139, 140). These patients already demonstrate spermatogonia loss before gonadotoxic treatment and adults have extremely low sperm concentrations (74, 139). For these reasons, fertility preservation in young Fanconi anemia patients undergoing HSCT is even more crucial.

### Others

Some other benign pathologies for which TT banking is recommended because of the fertility problems linked to high-risk treatment are inborn errors of metabolism for which other available therapies are less effective, and severe immune diseases that are not responsive to immunotherapy (11, 141). Well-known inborn errors of metabolism for which HSCT can be useful are lysosomal storage diseases, peroxisomal disorders, and mitochondrial diseases (141). Kostmann syndrome, chronic granulomatous disease, and Wiskott-Aldrich syndrome are some examples of immune diseases for which HSCT therapy may be applied (32, 142, 143). Additionally, high-risk agents such as cyclophosphamide are often used to suppress the immune system in patients with juvenile-onset systemic lupus erythematosus (144).

## Genital, Testicular, and Sexual Disorders

The third and last category of childhood conditions that may affect fertility and for which immature TT has been banked over the last years consists of genital, testicular, and sexual disorders (11). As this category contains markedly fewer patients than the previous ones, only the two most frequent indications will be explained in more detail.

### Cryptorchidism

Undescended testis or cryptorchidism is a pediatric condition in which one (unilateral cryptorchidism) or both testes (bilateral cryptorchidism) fail to descend into the scrotum. Cryptorchidism is one of the most common congenital anomalies that affects about 1-4% of full-term and 30% of preterm newborns worldwide (145). This condition is associated with hormonal defects, testicular torsion, inguinal hernia, and an increased risk of testicular germ cell tumors (146, 147). When the testes are absent in the scrotum during the first years of life, the number of germ cells will also drastically decrease, impairing future fertility (148). Previous research showed that around 13% of patients with unilateral cryptorchidism and up to 89% of patients with untreated bilateral cryptorchidism will be azoospermic later in life (149). Although the exact cause of the infertility and testicular malignancy is still unknown, researchers assume that the higher environmental temperature causes heat stress, resulting in abnormal transformation and excessive cell death of neonatal germ cells (149–151).

Some researchers suggested that hormone therapy with human chorionic gonadotrophin, GnRH, or LH-releasing hormone could have an additional protective effect on the fertility of boys with cryptorchidism. However, the results of these studies are not convincing and therefore, hormonal therapy is not advised (152). To decrease the risk of infertility and malignancy, orchidopexy is recommended to permanently anchor the testis into the scrotum. According to the American and European urology associations, this surgical procedure should be performed between 6-18 months of age as spontaneous testis descendance hardly occurs after 6 months of age (146, 153, 154). Besides this, later orchidopexy has been shown to reduce the testicular volume and the number of germ cells and to increase the risk of malignancy and infertility (154–157).

Nevertheless, the risk of azoospermia remains 18–46% in patients with bilateral cryptorchidism despite early surgical treatment (149, 158). To preserve the fertility of these patients while germ cells are still present in their testes, TT could be harvested for long-term storage during the orchidopexy procedure or during a separate procedure (92, 159, 160). This cryopreservation procedure is generally well accepted by the parents (161). Since patients with unilateral cryptorchidism generally have a low risk of becoming infertile, TT cryopreservation is not recommended. For patients with bilateral cryptorchidism, it is proposed to harvest TT during the orchidopexy procedure because they have a higher risk of later infertility. However, retrieving TT during a separate biopsy procedure is not recommended as this would require another invasive surgery procedure (160).

### Klinefelter Syndrome

With an incidence of as much as 1-2/1,000 newborn males, Klinefelter syndrome (KS) is the most frequent sex chromosome abnormality in the human (162). About 80% of the KS patients have a 47, XXY karyotype and the other 20% carry higher grade aneuploidies or mosaicisms (163). The phenotype of KS is highly variable and is generally worse in patients with a 47,XXY karyotype or higher-grade aneuploidies compared to mosaic patients (164). Typical KS symptoms are a tall and feminine posture, gynecomastia, neurocognitive and psychosocial problems, cryptorchidism, small firm testes, and hypogonadism (165, 166). Moreover, KS is characterized by germ cell loss already in early childhood and from puberty onwards testicular fibrosis, degeneration of the seminiferous tubules, and Leydig cell hyperplasia (167, 168). More than 90% of the non-mosaic KS patients are diagnosed with azoospermia later in life (169).

With this in mind, TESE during early adolescence has been proposed in the hope of having better success rates compared to TESE in adulthood (170–172). The rationale behind this was to perform the TESE procedure before the testicular tissue degeneration and before testosterone supplementation which could further suppress spermatogenesis (173). However, to this date, no proof shows that a biopsy during prepuberty or a TESE during adolescence is more successful than a TESE during adulthood (167, 173–175).

In order to perform fertility preservation before germ cell loss, KS patients have also been enrolled in several experimental immature TT banking programs (96, 167, 176, 177). However, a lot of the biopsied testicular tissues were found to be sclerotic, making fertility restoration methods, including auto-transplantation and *in vitro* spermatogenesis (IVS), unfeasible. Additionally, as the biopsies only rarely contained spermatogonia and the TT biopsy procedure may further reduce the number of spermatogonia, TT banking in KS patients remains a highly controversial subject (11, 167, 178). It is important to note that the children and adolescents included in these studies (96, 167, 176, 177) are a different population than the adults who were included in earlier TESE studies. It could therefore be possible that these children and adolescents also score poorly with a TESE when they are grown up. More longitudinal research is required to validate this.

As TESE outcomes at adulthood are comparable to those at adolescent age (179), it is reasonable to wait until the patient has a concrete childwish. Therefore, immature TT banking is not recommended for KS patients outside research programs, and TESE during early adulthood should remain the first line fertility preservation method (169, 180, 181).

### Others

Other developmental and acquired conditions such as testicular torsion, varicocele, reproductive tract infections, hypospadias, ambiguous genitalia, benign tumors, spina bifida, and congenital adrenal hyperplasia are also associated with male infertility (182). As most of these conditions can be surgically treated during childhood or adulthood, these patients generally do not face a high risk of infertility and immature TT is not frequently banked (11).

## Discussion

Since 2002, fertility centers are offering immature TT banking to young patients who are at high risk of losing their SSCs (22, 25, 159, 183–185). Initially, centers only cryopreserved TT from cancer patients at significant risk of infertility due to the gonadotoxic properties of chemo- and radiotherapy (10). Gradually, an increasing number of centers is offering fertility preservation, resulting in expanding inclusion criteria (11). However, as TT banking is still experimental and restoration methods are not yet clinically performed, it is of great importance that TT banking is currently only offered to patients at high risk of infertility and for whom the advantages of this experimental procedure outweigh its potential risks (11, 20). Therefore, this review provides advice on which pediatric patients could benefit from TT banking.

Concerning children with malignant diseases, previous research has shown that most parents agree to preserve TT, even with a low risk of later fertility problems and an unknown success rate of future fertility restoration techniques (21, 186–189). Despite these findings, we agree with the recent guidelines of the PanCareLIFE Consortium and the International Late Effects of Childhood Cancer Guideline Harmonization Group (70) and only recommend TT banking for young cancer patients undergoing high-risk gonadotoxic treatment. This high-risk group consists of patients who need to undergo high-dose alkylating or platinum-based agents, TBI, testicular radiotherapy, or a combination. In some cases, cancer patients do not receive high-risk therapy unless they show a weak response to first-line low-risk chemotherapy or after relapse (73, 97). Since previous findings have shown that the SSC pool remains within the normal range after the first-line low-risk treatment, these patients should also be offered TT cryopreservation before the initiation of the high-risk treatment (71, 74, 190). However, additional research should be performed to verify whether the quality of the SSCs is still sufficient for fertility restoration after chemotherapy. When considering methods of fertility restoration, the risk of contamination of the biopsy with malignant cells must also be taken into account. To avoid this, tissue auto-transplantation is not recommended, and SSC transplantation after depletion of the malignant cells should be the preferred method. Nonetheless, before the latter would be clinically applicable, SSC purification techniques need to be fine-tuned (11, 12). Alternatively, IVS followed by ICSI could be proposed, but this technique still requires extensive research before it can be applied in the clinic (11).

Because of the invasive and experimental nature of the procedure to date, immature TT banking is not recommended for cancer patients with a medium- to low-risk treatment (70). Importantly, Kanbar et al. recently confirmed that these patients generally maintain their fertility, making TT banking redundant (27). However, as chemotherapeutic agents are mostly administered in combination, it is difficult to assess the individual risk per agent (58, 67, 69). Moreover, most results are extrapolated from animal or adult studies, making additional research necessary to verify these risk classifications/dosages in children (67, 68, 75, 76, 191). Therefore, studies with human-relevant experimental systems such as human organoids (18) or human organotypic culture combined with xenografting (192), could be helpful tools to characterize the exact effects of a chemotherapeutic agent on prepubertal testicular tissue. Besides this, FDA-approved targeted cancer treatments are not expected to cause a high risk of infertility, but further studies on long-term reproductive effects are needed to confirm this (19).

Although our risk classification is based on the most recent data available in the literature, it should be handled with caution for the reasons mentioned above. Furthermore, the risk of developing infertility is dependent on several patient-related factors like the patients’ age, type of cancer, and comorbidities (193). Consequentially, TT preservation should be advised on an individual basis, especially for patients with complex treatment protocols (194). Several studies demonstrate that the patients and their parents want to be educated about experimental fertility options despite the stressful time at diagnosis (21, 195). Therefore, regardless of the risk of the therapy, clinicians should counsel the patients and their families and inform them as soon as possible about the estimated effects of the therapy on the fertility of the patient (6, 70). The clinicians should be open to discuss possible fertility preservation options and refer the patient and his parents to a reproductive specialist when needed (193). Multi-collaborative care pathways with well-informed oncologists and reproductive specialists are necessary to facilitate the decision-making and increase the patient referrals and acceptance rates of the banking procedure (11, 196).

Analogous to cancer patients, children with non-malignant conditions often need high-risk chemo- and/or radiotherapy as conditioning treatment before HSCT. For this reason, immature TT banking is recommended in this patient group. Moreover, in SCD, thalassemia, and certain BMF patients, the disease itself could already cause some fertility issues. Patients with a FOXP3 gene deficiency or Fanconi anemia were also found to already have a reduced SSC pool prior to gonadotoxic therapy, which makes them even more vulnerable to its toxic effects (74, 139). Besides this, patients with SCD very often receive long-term HU treatment, posing an additional risk for later infertility (197). As none of these patients risk malignant cell contamination, tissue transplantation would be the most promising method for fertility restoration (11, 12).

Patients who suffer from bilateral cryptorchidism still have a relatively high risk for fertility problems, even after early orchidopexy (149, 158). Consequently, TT could be harvested during their recommended orchidopexy procedure while their testes still contain a sufficient number of germ cells (160, 161). Nevertheless, performing a separate TT biopsy procedure for this is not recommended. For Klinefelter patients and patients with other testicular disorders, TT banking is not recommended. This is because most testicular disorders can be treated surgically during childhood, which reduces the risk of infertility. For these patients, the benefits do not outweigh the possible negative effects of a testicular biopsy (11). Although patients with KS do have a high risk of azoospermia during adulthood, taking a TT biopsy is currently not advised as the tissue is often sclerotic and only rarely contains spermatogonia (11, 167, 178). It has been recommended to wait until the patient wishes to have children, as TESE combined with intracytoplasmic sperm injection shows promising results (169, 179–181).

In conclusion, today, immature TT banking is classified as an invasive and experimental procedure whose long-term effects and clinical applications have yet to be thoroughly investigated. Therefore, immature TT banking is only recommended for young cancer patients who need to undergo high-risk chemo- and/or radiotherapy, regardless of earlier low-risk treatment. Likewise, TT banking is advised for patients who need high-risk conditioning therapy before HSCT/BMT to cure their non-malignant disorder (e.g., sickle cell disease, beta-thalassemia, and bone marrow failure). Patients undergoing medium- to low-risk therapy and their parents should be informed about the possible impact of the therapy on the reproductive function, but TT banking is not advised in these patients. It is proposed to retrieve a TT biopsy during orchidopexy in patients with bilateral cryptorchidism. For Klinefelter patients, however, TT banking is not recommended because it generally does not give better outcomes than a TESE later in adult life. Also for patients with other testicular disorders, a TT biopsy is not recommended as the benefits usually do not outweigh the risks.

## Author Contributions

ED performed the literature search and manuscript writing, while AB and EG contributed to the critical revision of this review. All authors were involved in the conception and design of this review and approved the submitted version.

## Funding

This review paper was written with financial support from the Research Programme of the Research Foundation-Flanders (FWO), Kom Op Tegen Kanker (KOTK), and Wetenschappelijk Fonds Willy Gepts (WFWG).

## Conflict of Interest

The authors declare that the research was conducted in the absence of any commercial or financial relationships that could be construed as a potential conflict of interest.

## Publisher’s Note

All claims expressed in this article are solely those of the authors and do not necessarily represent those of their affiliated organizations, or those of the publisher, the editors and the reviewers. Any product that may be evaluated in this article, or claim that may be made by its manufacturer, is not guaranteed or endorsed by the publisher.
